# Supplementary material for: Excessive alcohol consumption and binge drinking in college students
Source: PeerJ. 2022 May 4;10:e13368. doi: 10.7717/peerj.13368 (PMC9083527; doi:10.7717/peerj.13368)
Supplement: Supplemental Information 2 [file peerj-10-13368-s002.docx]

**ID** (identification number)

**Age:** quantitative

**Gender:** Females=0; Male=1

**Place of residence:** Others = 3; Shared apartment = 2; Family home = 1; independent = 0

**Maternal level of studies:** No studies=3; Primary=2; Secondary/ vocational training=1; University=0

**Paternal level of studies:** No studies=3; Primary=2; Secondary/ vocational training=1; University=0

**BD (Binge drinking):** Yes=1; No=0

**AUDIT_1 (How often do you have a drink containing alcohol?)**

Never=0; Monthly or less=1; 2 to 4 times a month=2; 2 to 3 times a week=3; 4 or more times a week=4

**AUDIT_2 (How many standard drinks containing alcohol do you have on a typical day when drinking?)**

1 to 2=0; 3 to 4=1; 5 to 6=2; 7 to 9=3; 10 or more=4

**AUDIT_3 (How often do you have six or more drinks on one occasion?)**

Never=0; Less than monthly=1; Monthly=2; Weekly=3; Daily or almost daily=4

**AUDIT_4 (During the past year, how often have you found that you were not able to stop drinking once you had started?)**

Never=0; Less than monthly=1; Monthly=2; Weekly=3; Daily or almost daily=4

**AUDIT_5 (During the past year, how often have you failed to do what was normally expected of you because of drinking?)**

Never=0; Less than monthly=1; Monthly=2; Weekly=3; Daily or almost daily=4

**AUDIT_6 (During the past year, how often have you needed a drink in the morning to get yourself going after a heavy drinking session?)**

Never=0; Less than monthly=1; Monthly=2; Weekly=3; Daily or almost daily=4

**AUDIT_7 (During the past year, how often have you had a feeling of guilt or remorse after drinking?)**

Never=0; Less than monthly=1; Monthly=2; Weekly=3; Daily or almost daily=4

**AUDIT_8 (During the past year, how often have you been unable to remember what happened the night before because you had been drinking?)**

Never=0; Less than monthly=1; Monthly=2; Weekly=3; Daily or almost daily=4

**AUDIT_9 (Have you or someone else been injured as a result of your drinking?)**

No=0; Yes, but not in the last year=2; Yes, during the last year=4

**AUDIT_10 (Has a relative or friend, doctor or other health worker been concerned about your drinking or suggested you cut down?)**

No=0; Yes, but not in the last year=2; Yes, during the last year=4

**AUDIT_DOM1:** quantitative

**AUDIT_DOM2:** quantitative

**AUDIT_DOM3:** quantitative

**AUDIT TOTAL:** quantitative
