# Supplementary material for: Excessive alcohol consumption and binge drinking in college students
Source: PeerJ. 2022 May 4;10:e13368. doi: 10.7717/peerj.13368 (PMC9083527; doi:10.7717/peerj.13368)
Supplement: Supplemental Information 3 [file peerj-10-13368-s003.pdf]

Sublicencia en BiblioPRO

Se le ha otorgado la sublicencia de *Test para Identificar los Trastornos del Uso de Alcohol*

Estado de la sublicencia: Otorgada el 20 de December de 2021 a las 08:12

Válida hasta: 1 de January de 2020 a las 23:59

Información sobre el pago

- **Precio de la sublicencia:**
  - **Base imponible:** 0 €
  - **IVA:** 21% (0 €)
  - **Total:** 0 €
- **Número de la factura:**
- **Fecha de la factura:** No emitida
- **Número de albarán interno:** 4892_2021-12-17-SUBL-350-25643
- **IBAN:**
- **SWIFT:**

Información sobre la sublicencia

- **Nombre:** Manuel Herrero Montes
- **Email:** herreromm@unican.es
- **País:** España
- **Fecha de solicitud:** 17 de December de 2021 a las 11:11
- **Número de administraciones:** 150
- **Financiación mayoritaria por una entidad:** Académica
- **Entidad desde la que solicita la sublicencia:** Universidad de Cantabria
- **Promotor del proyecto:** Manuel Herrero Montes

Información sobre el estudio

- **Nombre:** Alteraciones neuropsicológicas y factores asociados al patrón de consumo de alcohol binge drinking en estudiantes universitarios
- **Uso:** Diagnóstico de problemas de salud
- **Ojetivos:**
- **Período:** 2018-01-01 - 2020-01-01
- **Diseño:** Transversal
- **Población:** Estudiantes universitarios
- **Enfermedad o síntoma:** No indicado
- **Modo de administración:** Autoadministrado
- **Soporte técnico:** Papel impreso

Datos de facturación

- **Razón social:** No indicado
- **CIF:** No indicado
- **Dirección:** No indicado
- **Código postal:** No indicado
- **Población:** No indicado
- **País:** No indicado

- **Teléfono:** No indicado
- **Correo electrónico:** No indicado

Condiciones de la sublicencia

El LICENCIANTE, la Fundación IMIM (NIF G60072253), en adelante IMIM, con domicilio legal en el Edificio PRBB, en la Calle Aigüader número 88, 08003 de Barcelona, España concede esta SUBLICENCIA de uso del CUESTIONARIO indicado, manifestando tener esta potestad según el acuerdo firmado con los titulares de los derechos de autor del cuestionario.

La solicitud de esta sublicencia por parte del solicitante, en adelante el LICENCIATARIO, implica su total aceptación de los términos y condiciones indicadas como “Avisos Legales” en la página Web de BiblioPro “www.bibliopro.org” así como de los PACTOS indicados a continuación, a partir de la fecha de aceptación del presente Acuerdo por parte del LICENCIATARIO.

PACTOS

1. DEFINICIONES

MATERIALES: Se refiere a la documentación adicional al cuestionario, éste incluido, que pueda servir para la correcta utilización del instrumento, como el manual de uso o las normas de puntuación, y sobre los cuáles los titulares de derechos del cuestionario sean autores.

COPIAS APROBADAS: Las reproducciones del cuestionario que se realicen para su uso según los PACTOS aquí establecidos, deberán guardar el formato en el que se ha obtenido el CUESTIONARIO a través de la página de BiblioPro, y, en todo caso, deberá mantener la información relativa a los derechos de propiedad así como a la fuente por la que se ha conseguido el cuestionario.

SUBLICENCIA: Licencia no-exclusiva, no-transferible, no-sublicenciable, otorgada por IMIM al LICENCIATARIO y en nombre de los titulares de derechos de autor del cuestionario para permitir su uso, según las condiciones y pactos indicados en este documento y únicamente para la persona física o jurídica, plazo y uso indicados por el LICENCIATARIO en el momento de realizar la solicitud por vía telemática.

LICENCIATARIO: Persona física o jurídica cuyos datos han sido registrados por vía telemática como el solicitante de la presente sublicencia y que no podrán ser modificados para subrogar los pactos que aquí se establecen a favor de otra persona física o jurídica.

2. SUBLICENCIA

Mediante esta sublicencia, IMIM otorga al LICENCIATARIO el derecho de reproducción del CUESTIONARIO así como el uso de sus COPIAS APROBADAS únicamente para el uso indicado por el LICENCIATARIO en el momento de su solicitud.
El LICENCIATARIO no obtiene ningún otro derecho que el especificado en este PACTO 2. En particular, el LICENCIATARIO no obtiene derechos de propiedad intelectual ni autoría, y no puede digitalizar, distribuir, comercializar ni modificar los MATERIALES sin el expreso acuerdo de sus titulares.

IMIM y los TITULARES DE DERECHOS DE AUTOR no otorgan ningún permiso para el uso de su/s nombre/s o logos en cualquier otra forma que la específicamente indicada en esta SUBLICENCIA.

El LICENCIATARIO no asignará los derechos de esta SUBLICENCIA a ninguna otra entidad o persona física o jurídica que la expresamente indicada en el momento de la solicitud de la sublicencia.

3. PROTECCIÓN DE LOS DERECHOS DE PROPIEDAD INTELECTUAL

El LICENCIATARIO se compromete a tomar todas las medidas razonables necesarias para proteger la propiedad intelectual del CUESTIONARIO, no pudiendo alterar ni utilizar los ítems, palabras; ni traducir o modificar su contenido; reproducir, ni transmitir el contenido de esta documentación, a no ser que esté expresamente previsto en el presente documento.

El LICENCIATARIO se asegurará de que todas las copias del CUESTIONARIO incluyan la información relativa a los derechos de propiedad intelectual.

4. CONFIDENCIALIDAD Y MEDIDAS CAUTELARES

El LICENCIATARIO conviene en que los MATERIALES son activos valiosos cuyo valor se vería significativamente afectado por la distribución o el uso no autorizados de estos. El LICENCIATARIO se asegurará de que los MATERIALES no serán utilizados para fines no autorizados ni por personas no autorizadas e informará de inmediato su conocimiento sobre cualquier uso no autorizado de este tipo a BiblioPRO. El LICENCIATARIO conviene en que, en caso de cualquier incumplimiento del contenido de este párrafo por parte suya, las indemnizaciones monetarias no serán un recurso legal suficiente, y que la FIMIM, en la medida que lo permita la ley aplicable, tendrá derecho a una compensación igual a la máxima permitida por los recursos legales disponibles y equitativa.

5. EXCLUSIÓN DE GARANTÍAS

El LICENCIATARIO entiende y conviene en que, productos complejos y sofisticados como los MATERIALES están sujetos de forma inherente a defectos no descubiertos. IMIM no puede representar ni garantizar, y no representa ni garantiza, al LICENCIATARIO que los MATERIALES estén libres de dichos defectos, que funcionen sin interrupciones ni errores ni que los resultados obtenidos sean efectivos o apropiados en cualquier aplicación determinada. LOS BIENES Y SERVICIOS SE OFRECEN EN EL PRESENTE DOCUMENTO TAL Y COMO SON, Y IMIM NO HACE NINGÚN TIPO DE DECLARACIONES NI GARANTÍAS, EXPRESAS O IMPLÍCITAS, DERIVADAS DE LA LEY O DE ALGUNA OTRA FORMA, RELATIVAS A DICHOS BIENES, SERVICIOS O AL PRESENTE ACUERDO, Y SE DESLIGA DE TODO TIPO DE GARANTÍAS, INCLUIDAS, PERO NO LIMITADAS A, CUALQUIER DECLARACIÓN O GARANTÍA REFERENTE A COMERCIABILIDAD, IDONEIDAD PARA UN FIN CONCRETO, AUSENCIA DE VIOLACIÓN U OTRO ASPECTO.

6. LIMITACIÓN DE RESPONSABILIDAD

INDEPENDIENTEMENTE DE SI CUALQUIER RECURSO LEGAL DISPUESTO EN EL PRESENTE DOCUMENTO NO CUMPLE CON SU PROPÓSITO FUNDAMENTAL, LA RESPONSABILIDAD TOTAL DE IMIM SOBRE TODAS LAS RECLAMACIONES DERIVADAS DEL PRESENTE DOCUMENTO, NO EXCEDERÁ BAJO NINGUNA CIRCUNSTANCIA LA CUANTÍA DE LAS TASAS PAGADAS POR EL LICENCIATARIO POR EL USO DE LOS MATERIALES.

7. PLAZO DE VIGENCIA, MODIFICACIÓN Y CAUSAS DE EXTINCIÓN

La presente SUBLICENCIA entrará en vigor en el momento de su expedición por medios telemáticos al LICENCIATARIO, y permanecerá vigente hasta la fecha indicada por el LICENCIATARIO en el momento de la solicitud, pudiendo prorrogarse su uso con el acuerdo de BiblioPRO (IMIM), estableciéndose un límite máximo de cuatro años, prorrogables bajo petición expresa.

No obstante lo anterior, IMIM y/o los PROPIETARIOS INTELECTUALES podrán revocar los derechos otorgados al LICENCIATARIO a través de esta sublicencia, dando aviso de dicha decisión por escrito.

Los derechos otorgados en esta SUBLICENCIA se extinguirán de manera automática por:

a) La falta de cumplimiento de cualquiera de los PACTOS por parte del LICENCIATARIO
b) Imposibilidad sobrevenida del cumplimiento de las actividades descritas

La extinción de los derechos otorgados en esta SUBLICENCIA no conllevarán la extinción de las obligaciones suscritas por el LICENCIATARIO al solicitar dicha sub-licencia. En cualquier caso de terminación de esta sub-licencia, el LICENCIATARIO deberá devolver o destruir inmediatamente todas las copias de los MATERIALES, así como aportar pruebas de dicha destrucción, en caso de que IMIM se lo requiera

8. MISCELANEAS

Cualquier pregunta relacionada con esta SUBLICENCIA o con el CUESTIONARIO puede ser dirigida al equipo BiblioPro a través de los formularios de contacto habilitados en su página Web http://www.bibliopro.org, o bien a través del correo electrónico bibliopro@bibliopro.org.

La presente SUBLICENCIA contiene todo el acuerdo existente entre las partes y no hay otras representaciones, garantías, promesas o compromisos aparte de los contenidos en el presente acuerdo. Este documento prevalece y cancela todos los acuerdos previos existentes entre las partes.

La presente SUBLICENCIA se someterá a las leyes de España y todas las demandas derivadas del presente acuerdo serán presentadas en el Tribunal correspondiente de la ciudad de Barcelona, renunciando expresamente las partes a cualquier otra jurisdicción que pudiere corresponderles.

La presente SUBLICENCIA entra en vigor en el momento de su expedición por vía telemática por parte de IMIM, habiendo expresado su acuerdo explícito el LICENCIATARIO al solicitarla por vía telemática, de acuerdo con las condiciones legales establecidas en el sitio Web de BiblioPro, y IMIM por el simple hecho de expedirla a través de los medios telemáticos de BiblioPro.
